# Plasma osteopontin is a biomarker for the severity of alcoholic liver cirrhosis, not for hepatocellular carcinoma screening

**DOI:** 10.1186/s12876-015-0307-1

**Published:** 2015-06-30

**Authors:** Adélia Simão, João Madaleno, Nuno Silva, Fernando Rodrigues, Paula Caseiro, José Nascimento Costa, Armando Carvalho

**Affiliations:** 1 Internal Medicine A-Centro Hospitalar e Universitário de Coimbra, Portugal, and University of Coimbra, Faculty of Medicine, Coimbra, Portugal, Av. Bissaya Barreto e Praceta Mota Pinto, 3000-075 Coimbra, Portugal; 2 Clinical Pathology-Centro Hospitalar e Universitário de Coimbra, Coimbra, Portugal

## Abstract

**Background:**

Implementation of surveillance programs for at-risk populations and identification of biomarkers for early hepatocellular carcinoma (HCC) detection are a major public health goal. Recently, osteopontin (OPN) has attracted attention as a promising biomarker, with some potential advantages compared to alpha-fetoprotein (AFP), but its role in the context of alcoholic cirrhosis has never been assessed. The aims of this study are to assess the utility of plasma OPN in the diagnosis of HCC in alcoholic cirrhotic patients and to investigate whether increased values are due to the tumor or underlying liver disease severity.

**Methods:**

A total of 90 consecutively alcoholic cirrhosis patients, observed between Jun 2013 and May 2014 at a Liver Disease Unit, were included and divided into two groups: 45 without (group I) and 45 with HCC (group II). Plasma levels of OPN (ELISA, Immuno-Biological Laboratories, Gunma, Japan) and AFP (IMMULITE® 2000 AFP, Siemens Healthcare Diagnostics, Tarrytown, New York) were assessed. The diagnostic accuracy of each marker was evaluated using Receiver-Operating Characteristic (ROC) curve analysis (AUC) and its 95 % Confidence Interval (CI).

**Results:**

Plasma OPN levels in group I patients (1176.28 +/–744.59 ng/mL) weren’t significantly different from those of group II (1210.75 +/–800.60 ng/mL) (*p* = 0.826). OPN levels significantly increased with advancing BCLC tumor stage and with advancing Child-Pugh class, in both groups. Comparing the two groups, AUC for OPN and AFP were 0.51 (95 % CI: 0.39–0.63) and 0.79 (95 % CI: 0.70–0.89), respectively. Based on the ROC analysis, there were no satisfactory cut-off values for OPN that would distinguish patients with from those without tumour.

**Conclusions:**

Despite having a correlation with BCLC stage, the same was observed with progressive deterioration of underlying liver function in terms of Child-Pugh class and MELD score, and isn’t a useful diagnostic biomarker for HCC in alcoholic cirrhotic patients, particularly in the early stages. AFP confirms the performance evidenced in other studies, being superior to OPN. Searching more specific biomarkers for early diagnosis of HCC in alcoholic cirrhosis is still warranted.

## Background

Diagnosis of hepatocellular carcinoma (HCC) has recently increased in many low incidence countries including Portugal [10, 16, 37]. The incidence and mortality rates are heterogeneous, but mortality during the last decades increased and according to World Health Organization (WHO) has a mortality rate of about 47,000 deaths/year in Europe, close to the rate of incidence. Early detection of HCC opens doors for various effective treatments such as surgical resection and transplantation, which can subsequently lead to long-term survivals in a greater number of patients. Although surveillance and better diagnostic techniques have improved outcomes, the overall 5 years survival rate is only 8.6 % in Europe [7, 39].

In 80–90 % of cases, HCC develops on underlying liver cirrhosis or inflammation [21]. Worldwide, approximately 54 % of cases can be attributed to HBV infection while 31 % can be attributed to HCV infection, leaving approximately 15 % associated with other causes. The percentage of alcohol related HCCs is not well defined, but alcoholic cirrhosis is clearly a risk factor for HCC [15], and in one study, alcoholic liver disease accounted for 32 % of all HCC’s [22]. In Portugal, for instance, alcoholic cirrhosis (AC) is the most common cause of chronic liver disease being responsible for 84 % of a total of 81 543 hospital admissions for cirrhosis between 1993 and 2008 [31].

Implementation of surveillance programs to identify at-risk candidate populations and identification of biomarkers for early HCC detection are a major public health goal to decrease HCC-related deaths. According to the EASL–EORTC Clinical Practice Guidelines on Management of HCC, patients with cirrhosis are at high risk and should be enrolled in surveillance programs. The current recommendation for HCC surveillance consists of ultrasound that should be performed every 6 months in patients at high risk. This method has a sensitivity of 60 % [9, 28], although the presence of cirrhosis, with fibrous septa and regenerative nodules, produce a coarse pattern which may impair identification of small tumours. [14].

Alpha-fetoprotein (AFP) is the most widely used serological marker but has a suboptimal performance and is considered an inadequate screening test for HCC. The association of AFP and ultrasound significantly increases costs and the number of false positives, seeming to have no advantage in practice and therefore is not currently recommended [11, 13].

Other serological biomarkers have been or are under investigation for early diagnosis of HCC, including des-gamma-carboxy prothrombin (DCP) (also known as prothrombin induced by Vitamin K Absence II–PIVKA II), the ratio of glycosylated AFP (L3 fraction) to total AFP, alpha-fucosidase, and glypican 3. None of these have better performance characteristics than AFP.

Recently osteopontin (OPN) attracted attention as a promising biomarker for HCC diagnosis in patients with virus related cirrhosis with better sensitivity than AFP in differentiating HCC cases from cirrhosis controls as suggested by the results of two major studies [27, 36], which included patients with liver disease, particularly chronic HBV or HCV infections.

Kim J and coworkers determined plasma levels of OPN (ELISA), as well as AFP and PIVKA II, in 62 patients with HCC (69 % HBV, 10 % HCV, 3 % alcohol related), in 60 patients with chronic liver disease without tumor (83 % HBV, HCV 3 %, 10 % alcohol related) and in 60 healthy controls. Significantly higher plasma OPN levels (p <0.001) where detected in patients with HCC (median 954 ng/ml, range 168–5742) than in chronic liver disease (381 ng/ml, 29–1688) and healthy controls (155 ng/ml, 10–766). OPN levels correlated with progressive deterioration of underlying liver function in terms of Child-Pugh class and advancing degree of tumor stage. The sensitivity and specificity of OPN for the diagnosis of HCC were 87 % and 82 %, respectively, for a cut-off of 617.6 ng/mL. OPN had an AUC (0.898) greater than AFP (0.745) or PIVKA II (0.578), suggesting a better diagnostic accuracy. Immunohistochemistry showed OPN expression of in 92 of 285 tumors (32.3 %) and was found in malignant hepatocytes and macrophages, which invade the tumor, but not in normal hepatocytes or in Kupffer cells [27].

Shang S *et al.* performed proteomic profiles of plasma from patients with cirrhosis or HCC and validated selected candidate HCC biomarkers in two geographically distinct cohorts to include HCC of different etiologies. Mass spectrometry profiling identified OPN as significantly up-regulated in HCC cases. OPN levels were subsequently measured in 312 plasma samples from 131 patients with HCC, 76 cirrhotics, 52 with chronic hepatitis B or C and 53 healthy controls, belonging to two independent cohorts. OPN has a higher sensitivity than AFP in the diagnosis of HCC in all groups and was also useful in HCC patients with normal AFP. A prospective pilot study involving 22 patients who developed HCC during follow-up found that OPN was already increased one year before diagnosis, thus suggesting a potential predictive role of this biomarker for the occurrence of the tumor [36].

However, it is known that OPN correlates to other tumors and pathological conditions, which can impose a strong limitation to its use as a HCC marker. Indeed, some studies have demonstrated its role in tumorigenesis and metastasis formation, and expression of OPN has been detected in several types of carcinomas in humans. Despite that, OPN is an attractive potential tumor marker, found in the extra-cellular matrix secreted and also in body fluids, including plasma [25].

OPN expression is found physiological in bone and kidney, but can also be detected in many organs in pathological conditions. Hepatic expression of OPN was first found in Kupffer cells, stellate cells and macrophages in inflammatory and necrotic areas, in rats with carbon tetrachloride intoxication [25]. Subsequently, it was shown to have increased expression in patients with AC, and also in cirrhosis of other etiologies, like NASH, primary biliary cirrhosis, autoimmune hepatitis, primary sclerosing cholangitis, suggesting that chronic liver injury is the main factor for the induction of OPN response [38]. Serum OPN levels are correlated with hepatic inflammation and fibrosis in heavy alcohol drinkers, and hepatic OPN expression levels are strongly correlated with hepatic neutrophils accumulation, the pro-fibrogenic factor TGF-beta and fibrosis [5, 6, 19, 33].

Circulating levels of OPN are elevated in patients with liver lesions associated with HCV and HBV infections. For example, higher levels were an excellent indicator of cirrhosis in patients with chronic hepatitis B [40] and correlated with liver fibrosis in chronic hepatitis C, as found by Huang W *et al*. [23].

Although data suggests a better performance of plasma OPN in the diagnosis of HCC, the role of this biomarker needs validation. Moreover, data are lacking in alcoholic liver disease (only 2 in 62 patients with HCC have alcoholic cirrhosis in the study of Kim J et al.), the most common risk factor of HCC among us. So we conducted a study with the following objectives: (1) evaluate the usefulness of plasma OPN in the diagnosis of HCC in patients with alcoholic cirrhosis, and compare its accuracy with AFP; (2) investigate whether increased OPN is due to the tumor or underlying disease; and determine if there is any relationship between plasma OPN levels and the activity or severity of liver disease.

## Patients and methods

### Patients and plasma samples

This study was performed with approval from the Ethics Committee of the Faculty of Medicine of the University of Coimbra, and written informed consent was obtained from each patient. Patients followed between Jun 2013 and May 2014 at the Liver Disease Unit–Internal Medicine Department and Hepatic Transplantation Unit at Coimbra Hospital and University Centre were included. A total of 90 consecutively observed patients with AC were included and divided into two groups: group I included 45 patients with AC, and group II included 45 patients with AC and HCC.

The diagnosis of AC was established on the basis of clinical, laboratory, imaging (ultrasonography and computed tomography), transient elastography and histological examinations, as needed. All patients had a history of alcohol intake >60 g/day for more than 10 years and other causes of liver disease (HBV, HCV, autoimmune and metabolic diseases) were excluded. Status of liver function was defined as grades A, B or C based on the Child-Pugh classification [20] and Model for End-stage Liver Disease (MELD) Score was calculated [24].

The diagnosis of HCC was based according to the non invasive criteria of EASL–EORTC Clinical Practice Guidelines on Management of HCC. Tumor status, liver function and health status (ECOG) were obtained and patients were divided in stages (0, A, B, C and D) according to the Barcelona Clinic Liver Cancer (BCLC) staging system [12].

In all the subjects, blood was collected in a plastic tube containing ethylene diamine tetra acetic acid (EDTA), and isolated plasma and serum samples were stored at–80 °C until measurements of OPN and AFP levels. All measurements were performed at Immunology Unit of the same institution.

### Measurement of plasma OPN level

Plasma OPN levels were measured using an enzyme-linked immune sorbent assay (ELISA) kit (human osteopontin assay kit, Immuno-Biological Laboratories, Gunma, Japan) according to the manufacturer’s instructions. This ELISA kit measures total concentration of both phosphorylated and nonphosphorylated forms of OPN in plasma. All the experiments were performed in duplicate.

### Measurement of serum AFP Level

Serum AFP levels were measured with the same sample by the chemiluminescence method using IMMULITE® 2000 AFP kit (Siemens Healthcare Diagnostics, Tarrytown, New York) according to the manufacturer’s instructions.

### Statistical analysis

Median, range, mean, and standard deviation were used for descriptive statistics, as appropriate. Categorical variables were tested with Fisher’s exact test or *χ*^2^ test. Continuous variables were tested with Student *t-*test. Comparison of plasma OPN and AFP levels and clinical characteristics among the two groups of subjects were analyzed using the Mann–Whitney *U* test and Kruskal-Wallis test. Correlation between plasma levels of OPN and AFP were analyzed using Spearman’s correlation coefficient. Receiver operating characteristics (ROC) analysis was used to evaluate the diagnostic value of OPN, AFP, and to identify the optimal threshold values. *p*-value <0.05 was considered statistically significant.

## Results

The demographics, functional status of liver in terms of Child-Pugh class, MELD score, and tumor stage of the subject patients are summarized in Table 1.

**Table 1:** Clinical characteristics of cirrhotic patients with and without hepatocellular carcinoma (HCC)

	Group I	Group II	*p*-value^*^
	(Cirrhosis)	(Cirrhosis and HCC)	
N (%)	45 (50.0 %)	45 (50.0 %)	
Age	58.68 ± 10.76	64.64 ± 7.73	0.003
Sex (F/M)	(1/49)	(0/50)	
Child-Pugh class			0.052
A	20 (44.4 %)	21 (45.6 %)	
B	8 (17.8 %)	16 (35.6 %)	
C	17 (37.8 %)	8 (17.8 %)	
MELD score^a^	14.04 ± 5.56	12.71 ± 4.25	0.205
BCLC Stage^b^			
0		2 (4.4 %)	
A		19 (42.2 %)	
B		11 (24.4 %)	
C		7 (15.6 %)	
D		6 (13.3 %)	

^*^p - Value was calculated by using Student *T* test or *χ*2 test

^a^Model for End-stage Liver Disease score

^b^Barcelona Clinic Liver Cancer

### AFP and OPN levels in patients with and without HCC

**Fig. 1:**
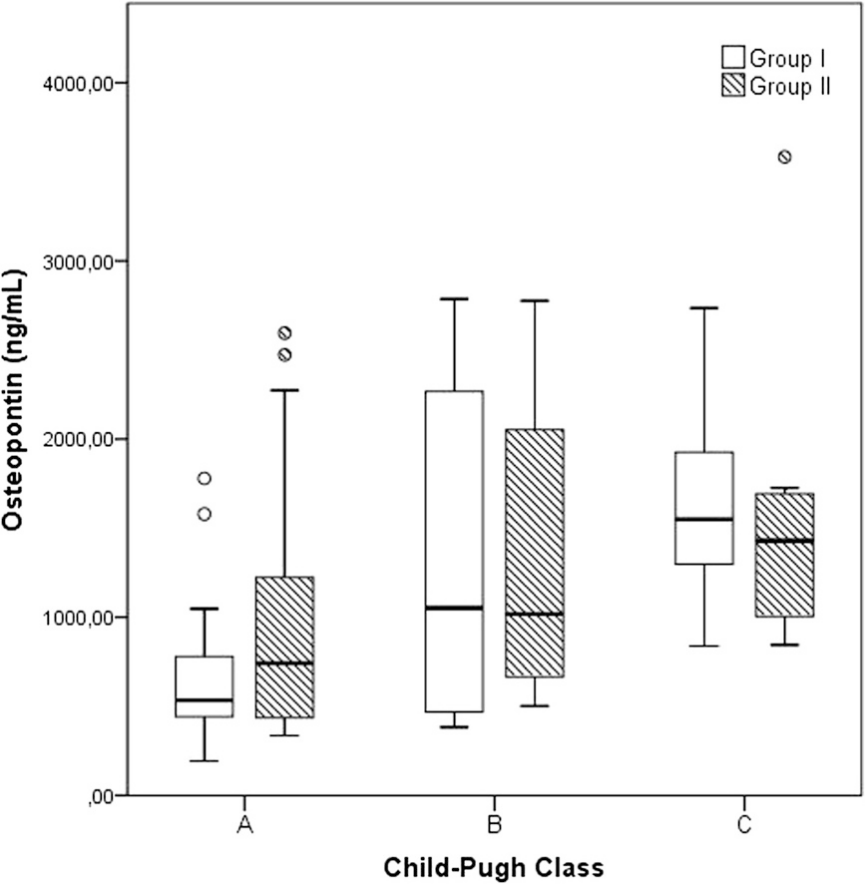
Plasma osteopontin (OPN) levels according to Child-Pugh class in patients with cirrhosis (Group I) and cirrhosis with hepatocellular carcinoma (Group II). Box refers to the 25th and 75th percentile values, with a line indicating median levels, whereas the interquartile range extends outside the box. Points outside the interquartile range are outliers. The plasma OPN level was progressively increased according to the Child-Pugh class in both groups

AFP and OPN levels in patients with and without HCC, and the distribution according to clinical features (Child-Pugh class and BCLC stage) are presented on Tables 2 and 3. AFP levels were significantly higher in cirrhotic patients with HCC than in those without HCC (*p*-value <0.001), but the same was not found with OPN, with no difference between groups (*p*-value = 0.826). Plasma OPN levels were progressively increased and are correlated with the degree of deterioration of functional liver status in terms of advanced Child-Pugh (Spearman rho [ρ] value of 0.533; *p*-value <0.001) and MELD score (Spearman rho [ρ] value of 0.518; *p*-value <0.001), but the same was observed regardless of the presence or absence of HCC as shown in Fig. 1. AFP serum levels were not correlated with the Child-Pugh class (ρ = 0.001; *p*-value = 0.990). Both OPN and AFP levels were significantly increased with advancing BCLC tumor staging. When we compared cirrhotic patients of group I with early stages of HCC (BCLC stages 0 and A), we found no difference between the two as determined by Mann–Whitney *U* test (*p*-value = 0.353).

**Table 2:** Serum levels of alpha-fetoprotein (AFP) in cirrhotic patients with and without HCC, and the relation to clinical features (Child-Pugh class and BCLC stage)

AFP (ng/mL)	Group I	Group II	*p*-value
	(Cirrhosis)	(Cirrhosis and HCC)	
	3.00 (0.90-17.00)	11.00 (1.30-431272.00)	<0.001^*^
Child-Pugh class			0.627 ^**^
A	2.50 (1.10-13.00)	10.00 (1.30-2159.00)	
B	3.40 (1.10-5.60)	14.00 (1.60-431272.00)	
C	3.10 (0.90-17.00)	12.45 (1.40-8251.00)	
BCLC Stage ^a^			0.007 ^**^
0		6.15 (5.90-6.40)	
A		3.20 (1.30-242.00)	
B		31.00 (2.80-431272.00)	
C		225.00 (8.70-7392.00)	
D		255.00 (3.30-8251.00)	

^*^p - Value by Mann–Whitney *U* test for comparison of serum AFP between two groups

^**^p - Value by Kruskal-Wallis test for comparison of serum AFP across Child-Pugh classes and BCLC stages

^a^Barcelona Clinic Liver Cancer

**Table 3:** Serum levels of osteopontin (OPN) in cirrhotic patients with and without HCC, and the relation to clinical features (Child Pugh class and BCLC stage)

OPN (ng/mL)	Group I	Group II	*p*-value
	(Cirrhosis)	(Cirrhosis and HCC)	
	923.80 (193.80-2786.20)	946.30 (337.10-3583.00)	0.826^*^
Child-Pugh class			<0.001^**^
A	534.95 (193.80-1778.60)	742.30 (337.10-2593.80)	
B	1051.85 (384.20-2786.20)	1017.80 (502.40-2775.10)	
C	1549.70 (838.40-2734.00)	1430.15 (844.00-3583.00)	
BCLC Stage^a^			0.025^**^
0		434.25 (394.40-474.10)	
A		844.00 (393.20-2775.10)	
B		742.30 (337.10-2472.00)	
C		1754.70 (363.80-2593.80)	
D		1609.75 (946.30-3583.00)	

^*^p-value by Mann–Whitney *U* test for comparison of plasma OPN between two group

^**^p-value by Kruskal-Wallis test for comparison of serum OPN across Child-Pugh classes and BCLC stages

^a^Barcelona Clinic Liver Cancer

### ROC analysis of OPN and AFP levels in patients with and without HCC

**Fig. 2:**
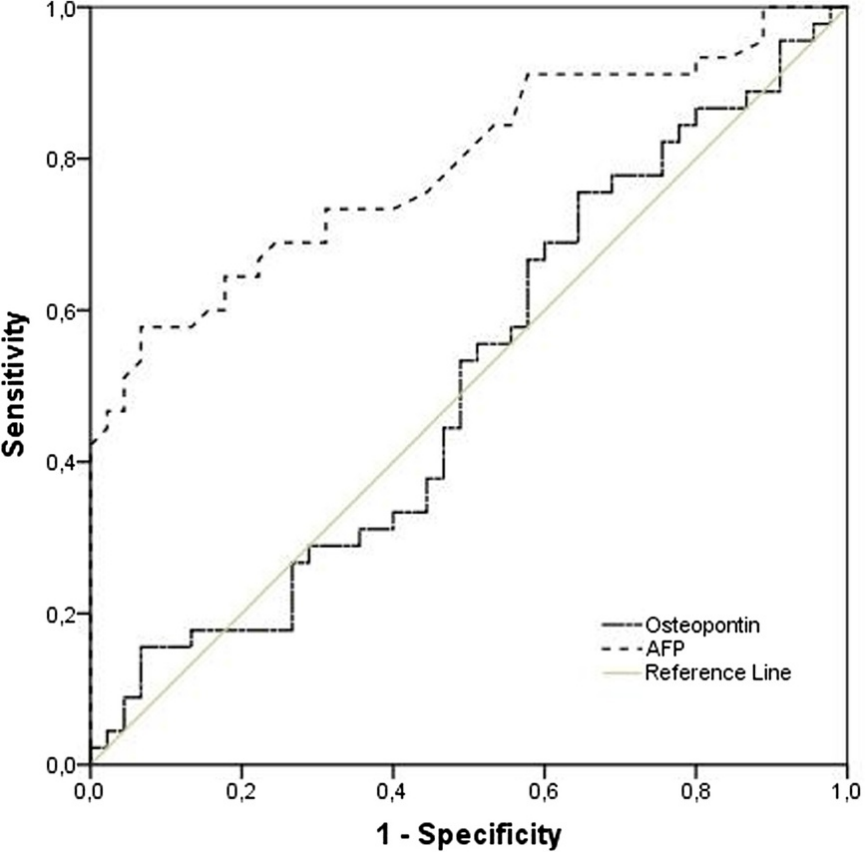
Receiver operating characteristics (ROC) curve for OPN (AUC = 0,51) and for AFP (AUC = 0,79)

Receiver operating characteristic (ROC) curves comparing for OPN and AFP are shown in Fig. 2. When HCC patients were compared to non HCC patients, the area under curve (AUC) for OPN (0.51; 95 % CI: 0.39–0.63) was lower than that of AFP (0.79; 95 % CI: 0.70–0.89), suggesting a non superior accuracy to AFP for the diagnosis of HCC. The sensitivity and specificity of AFP levels in HCC relative to AC group were 57.8 % and 93.3 %, respectively, at a cut-off value of 8.2 ng/mL (similar to the cut-off value recommended by manufacturer’s instructions). Based on the ROC analysis, there were no satisfactory cut-off values for OPN that would best distinguish HCC from non HCC patients.

## Discussion

In most published papers [3, 8, 27, 34–36] OPN showed an advantage over AFP in the diagnosis of HCC in patients with cirrhosis due to HBV or HCV infections, with the best performance obtained by combining OPN and AFP [36].

However, other studies published after the start of our work, have not confirm its usefulness in this context [1, 26] and until now there are no published data on the value of OPN in the diagnosis of HCC in patients with alcoholic liver cirrhosis. To our knowledge this is the first study of the role of OPN in this etiology of chronic liver disease.

In our study, plasma OPN levels were not significantly different between cirrhotic patients with and without HCC, with an AUC of 0.51 (95 % CI: 0.39 to 0.63), showing no significance as a diagnostic marker for the tumor.

The OPN value range and units are diverse in different studies, possibly due to the different techniques used. Kim J *et al*. used the same reference kits and the OPN values were of the same order of magnitude (average in the HCC group of 946.3 ng/mL and 964.0 ng/mL, respectively, in our patients and Kim J *et al.*).

OPN levels correlated with progressive deterioration of underlying liver function in terms of Child-Pugh class and MELD score in both groups, suggesting a correlation with clinical severity of liver disease. This favors the hypothesis that OPN is a protein with multiple functions implicated in hepatic inflammation and fibrosis, and could be a relevant biomarker for significant liver fibrosis [19, 33], but in our cohort all patients have liver cirrhosis and we do not have assessed OPN levels from individuals with non-cirrhotic alcoholic liver disease or from healthy subjects, so we cannot conclude about its use as a biomarker of fibrosis.

The difference between results of OPN in the chronic liver disease (CLD) group in the study of Kim J *et al.* (median 381 ng/mL; limits: 29–1688) and our (median: 946.30 ng/mL; limits: from 337.10 to 3583.00) may be due to the fact that they had only 50 % of cirrhosis in the CLD group, while in our case, all the patients had cirrhosis (and thus greater severity of liver disease). It is possible, of course, that the cause of CLD may also influence the plasma levels of OPN; in our study all patients were alcoholic, while in Kim J *et al.* only 10 % had this etiology.

In Group II we found a direct correlation of OPN with HCC BCLC stage, but our patients with early tumors (stages 0 and A), corresponding to 46.6 % of Group II patients, had average OPN values lower than that observed in Group I patients. Our data also shown that in alcoholic liver disease the amount of OPN is associated mainly with the severity of cirrhosis, and therefore could mask the increase that would occur due to the presence of HCC.

As already demonstrated in alcoholic patients, OPN correlates with hepatic inflammation, infiltration of neutrophils (alcoholic hepatitis), fibrosis and TGF-beta expression [19, 33]. In our patients we cannot prove that inflammation contributes significantly to the values of OPN, because none had clinical or analytical changes suggesting alcoholic hepatitis or other acute inflammatory process. However, we cannot categorically rule out this hypothesis, since we have no histological data from the time when blood draws for the OPN assays were collected.

According to the literature, the specificity of AFP for the diagnosis of HCC varies between 76 % and 96 %, being improved by raising the cut-off value, which in turn decreases sensitivity [18, 29]. AFP performance in our population was similar, with an AUC of 0.791 (95 % CI: 0.697 to 0.885), and sensitivity and specificity of, 55.6 % and 93.3 % respectively, for a cut-off 8.6 ng/ml (normal laboratory reference). Increasing the cut-off value to 23.5 ng/mL results in a specificity of 100 %, but a decrease in sensitivity to only 42.2 %.

A positive correlation between AFP serum levels and the stage of the tumor was shown in many studies [2, 17, 30, 32]. Similarly, in our patients we found the same correlation when we compared the serum AFP values with BCLC tumor stage. Moreover, AFP results were not influenced by the severity of liver cirrhosis, assessed by Child-Pugh score.

As previously said, we do not have histological data from the time when blood draws for OPN assays were collected, and in fact we only have liver biopsy in 26 patients (28,9 %), which is a limitation of our study and that’s why we could not exclude accompanying inflammatory processes, which deserves further research aimed to better characterize the role of alcoholic liver disease on the plasma levels of OPN. Another limitation was the sample size, as suggested by the wide range of OPN levels detected among patients with early stages of HCC patients. Although other causes of liver disease such as NAFLD or NASH were excluded, OPN levels are also increased in patients with type 2 diabetes, metabolic syndrome, obesity and smokers, but we cannot exclude these confounding factors since this was beyond the scope of the study and we haven’t performed this analysis. Finally, OPN is subject to alternative splicing as well as post-translational modifications, such as phosphorylation, glycosylation and proteolytic cleavage, and functional differences have been revealed for different isoforms and post-translational modifications. The pattern of isoform expression and post-translational modification is cell-type specific and may influence the potential role of OPN in malignancy and as a cancer biomarker, and some studies have reported that cleaved OPN may exhibit a better correlation with disease stage [4]. We used an ELISA kit that measures total concentration of both phosphorylated and nonphosphorylated forms of OPN in plasma, which can be a limitation of our study.

## Conclusions

In summary, the results of this study show that:

- OPN is not a suitable marker for HCC diagnosis in patients with liver cirrhosis of alcoholic etiology;
- In AC, OPN values increase with disease severity, regardless of the presence of HCC;
- In early stages of HCC (0 and A of BCLC classification) OPN value is lower than in patients without tumor, supporting the idea that the severity of cirrhosis is the cause of increased OPN in our population;
- In our patients, AFP confirms the performance evidenced in other studies, showing not to be the ideal marker, although being better than OPN.

These results confirm the lack of uselessness of tumor markers in HCC early diagnosis and reinforce the importance of radiological methods in the surveillance of risk groups, requiring, however, to be done by trained radiologists and proper equipment.

Finally, we emphasize that it is essential to continue research looking for new biomarkers, particularly important in AFP negative cases.

## Abbreviations

AFP: Alpha-fetoprotein
HCC: Hepatocellular carcinoma
OPN: Osteopontin
AC: Alcoholic cirrhosis
ROC: Receiver-operating characteristic
AUC: Area under the curve
CI: Confidence interval
BCLC: Barcelona clinic liver cancer
WHO: World health organization
HCV: Hepatitis C virus
EASL–EORTC: European Association for the Study of the Liver (EASL) and European Organisation for Research and Treatment of Cancer (EORTC)
DCP: Des-gamma-carboxy prothrombin
PIVKA II: Prothrombin induced by vitamin K absence II
HBV: Hepatitis B virus
NASH: Non alcoholic steato hepatitis
NAFLD: Non alcoholic fatty liver disease
MELD: Model for end-stage liver disease
EDTA: Ethylene diamine tetra acetic acid
ELISA: Enzyme-linked immune sorbent assay
CLD: Chronic liver disease

## References

[1] Abdel-Hamid M, Ellakwa DE, Omar NN (2014). Role of serum osteopontin level as a diagnostic biomarker for early hepatocelular carcinoma. Int J Cancer Res.

[2] Abelev GI, Perova SD, Khramkova NI, Postnikova ZA, Irlin IS (1963). Production of embryonal alpha-globulin by transplantable mouse hepatoma. Transplantation.

[3] Abu El Makarem MA, Abdel-Aleem A, Ali A, Saber R, Shatat M, Rahem DA (2011). Diagnostic significance of plasma osteopontin in hepatitis C vírus-related hepatocelular carcinoma. Ann Hepatology.

[4] Anborgh PH, Mutrie JC, Tuck AB, Chambers AF (2011). Pre- and post-translational regulation of osteopontin in cancer. J Cell Commun Signal.

[5] Apte UM, Banerjee A, McRee R, Wellberg E, Ramaiah SK (2005). Role of osteopontin in hepatic neutrophil infiltration during alcoholic steatohepatitis. Toxicol Appl Pharmacol.

[6] Banerjee A, Apte UM, Smith R, Ramaiah SK (2006). Higher neutrophil infiltration mediated by osteopontin is a likely contributing factor to the increased susceptibility of females to alcoholic liver disease. J Pathol.

[7] Berrino F, De Angelis R, Sant M, Rosso S, Lasota MB, Coebergh JW (2007). EUROCARE Working group. Survival for eight major cancers and all cancers combined for European adults diagnosed in 1995–99: results of the EUROCARE-4 study. Lancet Oncol.

[8] Bessa SS, Elwan NM, Suliman GA (2010). Clinical significance of plasma osteopontin level in Egyptian patients with hepatitis C virus-related hepatocellular carcinoma. Arch Med Res.

[9] Bolondi L (2003). Screening for hepatocellular carcinoma in cirrhosis. J Hepatol.

[10] Bosch FX, Ribes J, Diaz M, Cleries R (2004). Primary liver cancer: worldwide incidence and trends. Gastroenterology.

[11] Bruix J, Sherman M (2011). Management of hepatoclellular carcinoma: an update. Hepatology.

[12] Bruix J, Sherman M, Llovet JM, Beaugrand M, Lencioni R, Burroughs AK (2001). Clinical management of hepatocellular carcinoma. Conclusions of the Barcelona-2000 EASL conference. European Association for the Study of the Liver. J Hepatol.

[13] Clinical Practice Guidelines EASL-EORTC (2012). Management of hepatocelular carcinoma. J Hepatol.

[14] Colombo M (2007). Screening. Hepatol Res.

[15] Donato F, Tagger A, Gelatti U (2002). Alcohol and hepatocellular carcinoma: the effect of lifetime intake and hepatitis virus infections in men and women. Am J Epidemiol.

[16] Fattovich G, Stroffolini T, Zagni I, Donato F (2004). Hepatocellular carcinoma in cirrhosis: incidence and risk factors. Gastroenterology.

[17] Forner A, Reig M, Bruix J (2009). Alpha-fetoprotein for hepatocellular carcinoma diagnosis: the demise of a brilliant star. Gastroenterology.

[18] Gambarin-Gelwan M, Wolf DC, Shapiro R, Schwartz ME, Min AD (2000). Sensitivity of commonly available screening tests in detecting hepatocellular carcinoma in cirrhotic patients undergoing liver transplantation. Am J Gastroenterol.

[19] Gao B, Bataller R (2011). Alcoholic liver disease: pathogenesis and new therapeutic targets. Gastroenterology.

[20] Garcia-Tsao G, Bosch J (2010). Management of varices and variceal hemorrhage in cirrhosis. N Engl J Med.

[21] Hashem B, El-Serag (2011). Hepatocellular carcinoma. N Engl J Med.

[22] Hassan MM, Hwang LY, Hatten CJ, Swaim M, Li D, Abbruzzese JL (2002). Risk factors for hepatocellular carcinoma: synergism of alcohol with viral hepatitis and diabetes mellitus. Hepatology.

[23] Huang W, Zhu G, Huang M, Lou G, Liu Y (2010). Plasma osteopontin concentration correlates with the severity of hepatic fibrosis and inflammation in HCV-infected subjects. Clin Chim Acta.

[24] Kamath PS, Wiesner RH, Malinchoc M, Kremers W, Therneau TM, Kosberg CL (2001). A model to predict survival in patients with end-stage liver disease. Hepatology.

[25] Kawashima R, Mochida S, Matsui A, You LU, Tu ZY, Ishikawa K (1999). Expression of osteopontin in Kupffer cells and hepatic macrophages and Stellate cells in rat liver after carbon tetrachloride intoxication: a possible factor for macrophage migration into hepatic necrotic areas. Biochem Biophys Res Commun.

[26] Khalil A, Elgedawy J, Faramawi MF, Elfert A, Salama I, Abbass A (2013). Plasma osteopontin level as a diagnostic marker of hepatocellular carcinoma in patients with radiological evidence of focal hepatic lesions. Tumori.

[27] Kim J, Ki SS, Lee SD, Han CJ, Kim YC, Park SH (2006). Elevated plasma osteopontin levels in patients with hepatocellular carcinoma. Am J Gastroenterol.

[28] Kim CK, Lim JH, Lee WJ (2001). Detection of hepatocellular carcinomas and dysplastic nodules in cirrhotic liver: accuracy of ultrasonography in transplant patients. J Ultrasound Med.

[29] Kokudo N, Makuuchi M (2009). Evidence-based clinical practice guidelines for hepatocellular carcinoma in Japan: the J-HCC guidelines. J Gastroenterol.

[30] Margarit C, Charco R, Hidalgo E, Allende H, Castells L, Bilbao I (2002). Liver transplantation for malignant diseases: selection and pattern of recurrence. World J Surg.

[31] Marinho RT, Duarte H, Giria J, Nunes J, Ferreira A, Velosa J. The burden of alcoholism in fifteen years of liver cirrhosis hospital admissions in Portugal. Liver Int. 2014 doi: 10.1111/liv.12569. [Epub ahead of print] 10.1111/liv.12569 24750642

[32] Nomura F, Ohnishi K, Tanabe Y (1989). Clinical features and prognosis of hepatocellular carcinoma with reference to serum alpha-fetoprotein levels. Analysis of 606 patients. Cancer.

[33] Patouraux S, Bonnafous S, Voican CS, Anty R, Saint-Paul M-C (2012). The osteopontin level in liver, adipose tissue and serum is correlated with fibrosis in patients with alcoholic liver disease. PLoS One.

[34] Salem M, Sahar Abdel A, Maisa El R, Samar Kamal D, Marwa El S (2013). Clinical significance of plasma osteopontin level as a biomarker of hepatocellular carcinoma. Gastroenterology Res.

[35] Sawsan Said H, Aziza Ahmed El S, Manal Mohamed Abd AL A, Manal Abdel Baky M, Mohamed Omar El M, Mohamed Omar K (2013). Novel markers for the diagnosis of hepatocellular carcinoma. J Am Sci.

[36] Shang S, Plymoth A, Ge S, Feng Z, Rosen HR, Sangrajrang S (2012). Identification of osteopontin as a novel marker for early hepatocellular carcinoma. Hepatology.

[37] Sherman M (2005). Hepatocellular carcinoma: epidemiology, risk factors, and screening. Semin Liver Dis.

[38] Syn WK, Choi SS, Liaskou E, Karaca GF, Agboola KM (2011). Osteopontin is induced by hedgehog pathway activation and promotes fibrosis progression in nonalcoholic steatohepatitis. Hepatology.

[39] Verdecchia A, Francisci S, Brenner H, Gatta G, Micheli A, Mangone L (2007). EUROCARE-4 Working Group. Recent cancer survival in Europe: a 2000–02 period analysis of EUROCARE-4 data. Lancet Oncol.

[40] Zhao L, Li T, Wang Y, Pan Y, Ning H (2008). Elevated plasma osteopontin level is predictive of cirrhosis in patients with hepatitis B infection. Int J Clin Pract.

